# Selecting Essential Information for Biosurveillance—A Multi-Criteria Decision Analysis

**DOI:** 10.1371/journal.pone.0086601

**Published:** 2014-01-29

**Authors:** Nicholas Generous, Kristen J. Margevicius, Kirsten J. Taylor-McCabe, Mac Brown, W. Brent Daniel, Lauren Castro, Andrea Hengartner, Alina Deshpande

**Affiliations:** 1 Defense Systems and Analysis Division, Los Alamos National Laboratory, Los Alamos, New Mexico, United States of America; 2 Biosciences Division, Los Alamos National Laboratory, Los Alamos, New Mexico, United States of America; University of Vermont, United States of America

## Abstract

The National Strategy for Biosurveillancedefines biosurveillance as “the process of gathering, integrating, interpreting, and communicating essential information related to all-hazards threats or disease activity affecting human, animal, or plant health to achieve early detection and warning, contribute to overall situational awareness of the health aspects of an incident, and to enable better decision-making at all levels.” However, the strategy does not specify how “essential information” is to be identified and integrated into the current biosurveillance enterprise, or what the metrics qualify information as being “essential”. Thequestion of data stream identification and selection requires a structured methodology that can systematically evaluate the tradeoffs between the many criteria that need to be taken in account. Multi-Attribute Utility Theory, a type of multi-criteria decision analysis, can provide a well-defined, structured approach that can offer solutions to this problem. While the use of Multi-Attribute Utility Theoryas a practical method to apply formal scientific decision theoretical approaches to complex, multi-criteria problems has been demonstrated in a variety of fields, this method has never been applied to decision support in biosurveillance.We have developed a formalized decision support analytic framework that can facilitate identification of “essential information” for use in biosurveillance systems or processes and we offer this framework to the global BSV community as a tool for optimizing the BSV enterprise. To demonstrate utility, we applied the framework to the problem of evaluating data streams for use in an integrated global infectious disease surveillance system.

## Introduction

As defined in the National Strategy [1], biosurveillance is “the process of gathering, integrating, interpreting, and communicating essential information related to all-hazards threats or disease activity affecting human, animal, or plant health to achieve early detection and warning, contribute to overall situational awareness of the health aspects of an incident, and to enable better decision-making at all levels.” The systems and processes that constitute the biosurveillance (BSV) enterprise rely on a wide range of data that encompass human, animal, and plant health. An approach to enhancing biosurveillance capability is to increase the variety and range of data sources that are gathered, analyzed, and interpreted. Through the inclusionof new data typesit is possible to enhance existing surveillance systems as well as develop new and improvedversions. However, the inclusion and integration of newdata streams iscomplicated by a multitude of factors, such as the sheer diversity of potential data streams, the technical specifications and limitations of a system, financial constraints of system operators, etc. Building capability in this manner requires significant investments of technical, financial and human resources.

There is a recognized need for better methods and techniques within the biosurveillance community that would enable practitioners and system developers to prioritize and select the ‘best’ data streams for a biosurveillance system's specific intended use. In part, this is due to a lack of reliable and tested evaluation methods and criteria for evaluation. This presents a major hurdle to improving the efficiency of biosurveillance systems [2], [3], one which this team set out to address.

We used Multi-Attribute Utility Theory (MAUT), a type of multi-criteria decision analysis (MCDA), to develop ananalytic framework for biosurveillance data stream evaluation. MAUT and, more broadly, MCDA has been applied to assist decision makers with evaluation in a variety of fields that range from healthcare policyto power plant risk and urban planning [7]–[10]. The evaluation of biosurveillance data streams is a natural applicationfor MAUT.

MAUT is both an approach and a technique to analyze complex problems that produces a ranked list of prioritized options, also known as decision alternatives. It is a systematic approach to structuring a complex decision model where the decision alternatives include tradeoffs between costs and benefits. It simulates the decision making process by aggregating multiple single utility functions that each describe a certain facet of a decision alternative. The final utility score of an alternative is defined as the weighted sum of its single utility functions [4], [5]. The alternatives can then be ranked according to their final utility score, providing decision makers with a ranked list of prioritized decision alternativeswithunderlying assumptions and uncertainties explicitly defined.MAUT can also consider both quantitative and qualitative indicators as part of its analysis, a unique feature not frequently found in other types of evaluations. Ultimately, MAUT assists a decision maker in understanding the options available for solving a problem when the options presented have multiple attributes and where there is clearly no obvious best solution [6].

In the area of selection of data streams for BSV, there is a need to have a method that can systematically analyze the benefits and disadvantages of a data stream andby framing the question of biosurveillance data stream inclusion using MAUT, it is possible to build an evaluation framework that could be deployed for use by members of the biosurveillance enterprise.

While there have been no previous attempts identified to build an evaluation framework using MAUT for biosurveillance data streams, there are several studies that evaluate specific data streams using single a metric as well as a sizeable literature on the evaluation of biosurveillance systems [11]–[15]. Generic frameworks have been developed but they typically focus on certain categoriesor types of systemssuch as the utility of public health syndromic surveillance systems indetecting terrorist attacks [11] or for the evaluation of automated detection algorithms [12], [13]. Given the need of biosurveillance to consider disease activity across animal, plant, and human health, the applicability of many of these evaluation frameworks beyond their particular scope is limited.

Common methods of evaluation of biosurveillance systems use quantitative approaches that generally address only one or two attributes of a surveillance system [14]. Qualitative approaches of attributes of these surveillance systems are applied much less frequently. Another common approach is theestimationof the relative sensitivities obtained through comparison amongst one or more biosurveillance systems.While many different attributes such as sensitivity, accessibility, timeliness, etc. have been identified as being important [11], [14], few evaluations could be considered comprehensive (i.e. assess more than one or two attributes) [14], [15]. It is impossible for a single attribute to capture the full spectrum of criteria needed to make a robust evaluation. Even for evaluations that do describe multiple attributes, how the attributes are integrated or judged important israrely commented on [11].

Another characteristic lacking in traditional evaluation methods of biosurveillance systemsis the absence of context by which the system is being evaluated. Without understanding the goal, which in the case of biosurveillance can range from early detection of an outbreak to determining the effects of an outbreak control policy (such as vaccination), it is difficult to clearly define relevant measurableevaluative criteria. Evaluation methods that donot explicitly describe the context of evaluation weaken the rationale for selecting one attribute over because the evaluation metrics may change depending on the purpose of the biosurveillance system. The same metrics that are important for early detection (e.g. timeliness, time to detection, etc.) may not be as useful for another goal such as consequence management.Both the use of few attributes and the lack of explicitly defined biosurveillance objectives represent significant barriers to effective evaluation. Without these issues being addressed, it is not possible to have an unbiased and completeevaluation framework.

Using MAUT, we introduce a universal framework for evaluating biosurveillance data streams that can assess multiple attributes, both quantitative and qualitative, and that linksthese attributes to a specific (or defined) biosurveillance objective in order to provide a comprehensive and robust evaluation. By focusing on data streams used by biosurveillance systems, rather than evaluating the surveillance system itself, the framework becomes more universal in its application.This framework can be applied by biosurveillance practitioners, regardless of health domain, to assist in prioritizing the selection of data streams for inclusion into their surveillance program or system.

To demonstrate the utility of this framework, broad categories of biosurveillance data streams were evaluated. While the application of MAUT to evaluate specific data streams is the ultimate goal of the framework, this paper focused on broad categories of data streams in order to focus on the development of the framework (e.g. biosurveillance goals, metrics, decision criteria, etc.), thus laying the foundation for its eventual application to specific data stream evaluation. Having a tested, robust decision framework can assist practitioners and system developers in prioritizing the selection of data streams for inclusion in biosurveillance systems, thereby assisting in making valid, consistent, and justifiable programmatic decisions.

Proof of principle for the developed decision criteria framework is demonstrated by showing its applicability towards the evaluation of broad categories of data streams for inclusion in an integrated global infectious disease surveillance system.

## Methodology

### MAUT

Multi-attribute utility theory is a structured methodology that can calculate the overall desirability of an alternative in a single number thatrepresents the utility of that alternative. Theoverall desirability or utility of an alternative is calculated by the weighted sums of its measures (i.e. evaluation criteria). It is described by the following equation:
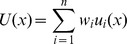
Where U(x) is the overall utility score for the alternative X, n is the number of measures, *w* is the relative importance of the metric, and u_i_(x) is the score of alternative X on the *i*th metric, standardized in a scale from 0 to 1 [5].

The theoretical framework of MAUT relies on several assumptions: that the decision maker prefers more utility over less utility, that the decision maker has perfect knowledge about what is being evaluated, that the decision maker is consistent in his/her judgments, and that the evaluation criteria are independent from one another.

The commercially available software package Logical Decisions (LDW) [16] was used to implement MAUT for this project. While the implementation methodology was developed around the input requirements of the software, the MAUT framework can be used with a simple spreadsheet if needed and is therefore agnostic to the tool used, making it universal.

### Development of Evaluation Framework

Our approach to the evaluation of data streams followed four broad stages—problem structuring, value elicitation, ranking, and sensitivity analysis—that could be sub-divided into seven steps, each of which were critically important to ensuring high confidence in our rankings (Table 1). The seven steps are described in the following paragraphs.

**Table 1:** EvaluationApproach.

Problem Structuring	1. BSV Goal and Objectives Identification
	2. Data Stream Identification
	3. Metric Identification
Value Elicitation	4. Metric Weight Assignment
	5. Value Assignment for Data Streams
Ranking	6. Data Stream Ranking
Sensitivity Analysis	7. Sensitivity Analysis

Under the problem structuring step, identification of biosurveillance goals, objectives, data streams, and metrics were identified through three approaches: a review of local, national and international surveillance systems, consultation with subject matter experts (SME), and areview of literature [17], [26]. The SME panel consisted of experts in human, animal, and plant health who worked in different sectors of biosurveillance (e.g. military, civilian, local, international, etc.). Only contact and affiliation information was collected about the individual SME responding to the questionnaire, and the survey was strictly a means to record expert opinion. Therefore, this survey did not involve human subjects research, and institutional review of the survey was deemed unnecessary (Common Rule(45 CFR 46), LANL Human Subjects Research Review Board (HSRRB)).

### 1. Identification of biosurveillance goals and objectives

Without describing the goals and objectives of biosurveillance in detail, it is not possible to structure an analysis framework and determine the relationship between evaluation criteria and the surveillance aims. Additionally, the prioritization and weighting of the different evaluation criteria are likely to differ depending on biosurveillance objectives.

We developed four BSV goals relevant for integrated global biosurveillance. Data streams wereevaluated for each goal separately. With this approach, it was possible to identify data streams that whilenot useful for one goal, were highly relevant for another. The four goals are arranged over a time scale that extends from pre-event to post-event (the event being a disease outbreak) regardless of origin. Data stream categories were evaluated for each of the following broad surveillance goals:

1. **Early Warning of Health Threats**: Surveillance that enables identification of potential threats including emerging and re-emerging diseases that may be undefined or unexpected.
2. **Early Detection of Health Events**: Surveillance that enables identification of disease outbreaks (either natural or intentional in origin), or events that have occurred, before they become significant.
3. **Situational Awareness**: Surveillance that monitors the location, magnitude, and spread of an outbreak or event once it has occurred.
4. **Consequence Management**: Surveillance that assesses impacts and determines response to an outbreak or an event

The overarching objective in our evaluation framework was to determine the most useful data stream(s) for each of these biosurveillance goals.

### 2. Selection of biosurveillance data streams

Determining the most relevant data streams is highly dependent on the biosurveillance objective. While the data streams identified should relate to the objective being considered, there is no need to limit the choice of data streams to a single type of data as it is important to assess the full range of possible data streams that may be useful for accomplishing the objective.

While we identified several hundred *specific* data streams that could be evaluated with the framework, it would have been impractical and of limited value to generate a prioritized list of several hundred data streams. Rather, webinned the data streams into broader categories/types of data streams and evaluated these categories in order to provide a moreusefuland informative result.Sixteen data steam categories were developed as shown in Table 2 [17]. This approach requireda level of detail that struck a balance between being too specific and too broadand allowed us realistic data set sizes for initial studies. However, a more in-depth data stream analysis couldbe performed using the same framework we have developed.

**Table 2:** Data Streams.

Data Stream	Definition
**Ambulance/EMT Records**	Dispatch information which can include incident date, time, nature of call, and patient information
**Clinic/Health Care Provider Records**	Record of patient (animal/human) information that can include symptoms, pharmacy orders, diagnoses, laboratory tests ordered and results received
**ED/Hospital Records**	Record of patient information that can include discharge/transfer orders, pharmacy orders, radiology results, laboratory results and any other data from ancillary services or provider notes
**Employment/School Records**	Information collected from schools or places of employment that can include, location, illness, absence and activity reports regarding students or employees
**Established Databases**	Any data repository from which information can be retrieved
**Financial Records**	Records of financial activities of a person, business, or organization
**Help Lines**	Telephone or cellular call-in services
**Internet Search Queries**	Search terms that a user enters into a web search engine
**Laboratory Records**	Information regarding specific tests ordered and/or the results of those tests
**News Aggregators**	Systematic collection of information from news sources that can include online and offline media
**Official Reports**	Any report that has been certified or validated from an authorized entity
**Police/Fire Department Records**	Dispatch and event information
**Personal Communication**	Any type of information that is directly relayed from one individual to another individual or group
**Prediction Markets**	Marketplaces for contracts in which the payoffs depend on the outcome of a future event
**Sales**	Monetary transactions for goods or services
**Social Media**	Forms of electronic communication such as websites for social networking and blogging through which users create online communities to share

### 3. Selection of Metrics

Metrics are the attributes, evaluation criteria, or measuresby which data streams are assessed. They should be carefully selected to be complete, measurable, mutually independent, and non-redundant. There is no optimal number of measures and the number will likely depend on the biosurveillance goal. However, if too few measures are chosen the results of the evaluation are likely not comprehensive. Conversely, if too many are chosen, it may needlessly complicate the analysis without necessarily leading to more useful results. A balance needs to be achievedbetween these two factors.

Similar to the objective formulation, we identified and selected measures (metrics) using a systematic and iterative process. It is important to note that unlike most of the previous literature, this project focused on describing metrics that would be used for evaluation of data streams not surveillance systems. Furthermore, because we evaluated data streams at a higher category level, many common metrics used to assess systems, such as positive predictive value, negative predictive value, sensitivity, and specificity etc. were not applicable [18]–[20].

Table 3 shows the list of 11 metrics developed by us for the evaluation of biosurveillance data streams. The table also provides definitions for each metric.

**Table 3:** Metrics and their Definition.

Metric	Definition
**Accessibility**	The extent to which the data stream is available
**Cost**	The cost to set-up, operate, and maintain the data stream
**Credibility**	The extent to which the data stream is considered reliable and accurate
**Flexibility**	The data stream's ability to be used for more than one purpose (such as for use in surveillance for more than one disease, or for more than one goal, etc.)
**Integrability**	How well the data stream can be linked/combined with other data streams
**Geographic/Population Coverage**	The geographic or population area of coverage
**Granularity**	The level of detail of the data stream
**Specificity of Detection**	The ability of the data stream to identify an outbreak, event, disease, or pathogen of interest
**Sustainability**	The data stream's continued availability over time
**Time to Indication**	The time required for the data stream to first signal a disease, outbreak, or event
**Timeliness**	Earliest time that the data is available

These metrics and definitions were used and refined throughout the process of the evaluation of the data streams. Every effort was made to develop metrics that would assess unique features of a data stream and would not overlap. However, it was clear that many of the metrics might have some level ofinterdependency. For example, cost andaccessibility are likely to be related—the cheaper it is to access that data stream, the higher the accessibility. A similar correlation exists between credibility and timeliness—the more quickly the data is available, the less likely it is credible. This interdependency could not be captured in the tool that we employed for evaluation.

When identifying the measures that describe the data streams, it was also important to determine how they could be measured because each measure needs to be described by a single utility function that describes the relationship between the value input and the utility the input provides towards achieving the goal. The values can be either quantitative or qualitative and the relationship between the value and utility needs to be explicitly defined. If the values are qualitative, concrete indicators need to be developed so that the data streams can be uniformly assessed. For example, we developed descriptions for measuring or assigning values to accessibility with three options that have specific criteria:

**Difficult Accessibility**—is when the data stream being analyzed has been used in at least one system and faces many (3 or more) obstacles in data access
**Medium Accessibility**—is when the data stream being analyzed has been used in at least one system and faces some (less than 3) obstacles in data access
**Easy Accessibility**—is when the data from a particular data stream is freely accessible.

Examples of obstacles include: privacy concern, passwords, subscription, membership/group affiliation, non-digitized information, etc. Table 4 displays the utility scores and labels that were used to describe the metrics. MAUT converts the values input for each metric to a common unit termed *utility*. It is important to note that the common unit *utility* is not the same as measuring utility (i.e. the “usefulness” of something). *Utility* is the unit that MAUT measures and works with in order to determine the overall utility (usefulness) of each alternative (data stream) from the evaluation criteria (metrics). Additionally, the relationship between *utility* and the values input for the criteria need to be defined (a utility function). For example, if the metric is cost, then the *utility* will decrease as the cost increases. The values can be specified as a quantity as well as by labels, which are text descriptions of the possible levels for each metric. Supplementary methods S1 contains information on the criteria used to assess the qualitative labels forthe metrics.

**Table 4:** Utility Scores for Metric Values.

Metric	Label	Utility Score
Accessibility	Easy	1
	Medium	0.5
	Difficult	0
Cost	High	0
	Medium	0.5
	Low	1
Credibility	High	1
	Medium	0.5
	Low	0
Flexibility	High	1
	Medium	0.5
	Low	0
Geographic/Population Coverage	Global	1
	National	0.667
	Regional	0.333
	Local	0
Granularity	Individual	1
	Community	0.667
	Regional	0.333
	National	0
Integrability	Extremely	1
	Highly	0.667
	Moderately	0.333
	Not Very	0
Specificity of Detection	High	1
	Medium	0.667
	Low	0.333
	Indirect	0
Sustainability	Yes	1
	No	0
Time to Indication	Long	0.333
	Medium	0.667
	Near Real Time	1
	Indirect	0
Timeliness	Slow	0
	Intermediate	0.333
	Fast	0.667
	Near Real Time	1

### 4. Assignment of Metric Weights

Not all metrics contribute equally to the utility of the data stream. Weights are assigned to metrics and can be used to define the relative importance of the metric towards achieving the biosurveillance goal. Many methods can be used to assign weights to metrics. Weights can be established via group discussion and deliberation, expert elicitation, or even direct rating of measures. To assign weights to the 11 metrics developed for the evaluation, weconsulted our SME panel and asked them to rank the metrics from 1 to 11 in order of importance for each objective. Definitions of the metrics and biosurveillance goal were provided and each SME was asked to rank according to the definitions provided (Table 3, Table 5). This approach reduced the possibility of individual biases in weighting based on one's interpretation of the terms. From the lists generated by the SMEs, the average rank of each metric was used to generate a priority list for each goal,.

**Table 5:** Definitions Provided to SME for the Metric Weight Survey.

**Question 1:** Rank these metrics in order of importance for use in an integrated global bio-surveillance system whose goal emphasizes Early Warning of Health Threats. Click on the Metric to drag and drop it in the order of importance 1 is most important and at the top, 11 is least important and at the bottom. Early Warning of Health Threats is defined as surveillance that enables identification of potential threats, including emerging and re-emerging diseases, that may be undefined or unexpected.
**Question 2:** Rank these metrics in order of importance for use in an integrated global bio-surveillance system whose goal emphasizes Early Detection of Health Events. Click on the Metric to drag and drop it in the order of importance 1 is most important and at the top, 11 is least important and at the bottom. Early Detection of Health Events is defined as surveillance that enables detection of disease, outbreaks (either natural or intentional in origin) or events that have occurred but are not yet identified.
**Question 3:** Rank these metrics in order of importance for use in an integrated global bio-surveillance system whose goal emphasizes Situational Awareness. Click on the Metric to drag and drop it in the order of importance 1 is most important and at the top, 11 is least important and at the bottom. Situational Awareness is defined as surveillance that monitors the location, magnitude, and spread of an outbreak or event.
**Question 4:** Rank these metrics in order of importance for use in an integrated global bio-surveillance system whose goal emphasizes Consequence Management. Click on the Metric to drag and drop it in the order of importance 1 is most important and at the top, 11 is least important and at the bottom. Consequence Management is defined as Surveillance that assesses impacts and determines response to an outbreak or event.

The rankings were then converted into metric weights using a mathematical technique called swing weighting, which is used in Simple Multi-Attribute Rating Technique Extended to Ranking (SMARTER) [16], [21]. By knowing the rank of the metrics, setting the value for the sum of weights to be 1 and giving equal weights to metrics if the preference is the same (i.e. if multiple metrics are ranked the same), the weights can be derived for each metric. Table 6 shows the weights derived for the metrics.

**Table 6:** Rankings of Metric Importance.

Early Warning of Health Threats		Early Detection of Health Events		Situational Awareness		Consequence Management	
1. Time to Indication	0.288	1. Time to Indication	0.275	1. Credibility	0.275	1. Credibility	0.271
2. Timeliness	0.188	2. Timeliness	0.184	2. Geo./Pop. Coverage	0.184	2. Geo./Pop. Coverage	0.146
3. Credibility	0.138	3. Credibility	0.138	3. Timeliness	0.138	2. Timeliness	0.146
4. Specificity of Detection	0.104	4. Specificity of Detection	0.108	4. Time to Indication	0.108	4. Specificity of Detection	0.105
5. Accessibility	0.079	5. Geo./Pop. Coverage	0.085	5. Accessibility	0.085	4. Time to Indication	0.105
6. Geo./Pop. Coverage	0.059	6. Accessibility	0.067	6. Specificity of Detection	0.067	6. Granularity	0.08
7. Flexibility	0.043	7. Granularity	0.052	7. Sustainability	0.052	7. Accessibility	0.059
7. Granularity	0.043	8. Integrability	0.039	8. Flexibility	0.039	8. Flexibility	0.041
9. Integrability	0.03	9. Flexibility	0.027	9. Integrability	0.027	9. Integrability	0.025
10. Sustainability	0.019	10. Sustainability	0.017	10. Granularity	0.017	10. Cost	0.011
11. Cost	0.009	11. Cost	0.008	11. Cost	0.008	10. Sustainability	0.011

### 5. Collection of Information – assignment of values to alternatives

As we evaluated data stream categories instead of individual, specific data streams,we faced a challenge with assigning values for each of the metrics for categories. To address this challenge,wefocused on the properties of data streams that were functional within operational biosurveillance systems, tools, or organizations, preferably global ones. The underlying assumption was that the individual, specific data streams within these systems were representative of the data stream category as a whole. This approach then could derive results that were grounded within the operational context of data streams within current surveillance systems, and while not ideal, allowed for the problem to be structured in a way that would yield meaningful results for development of the MAUT methodology of biosurveillance. For several data stream categories, we looked at more than one surveillance system to inform our assignment of values. Table 7 identifies the surveillance systems used to represent the data stream category. It is important to note that because not all data streams binned in the category would have these representative metric values, the results cannot be used to understand a specific data stream. Our approach was to use the categories to develop the framework for an initial top-level comparison of data streams in order to see if MAUT could be applied to biosurveillance data streams, paving the way for understanding how to use MAUT to evaluate specific data streams.

**Table 7:** Data Stream Categories and Representative Biosurveillance Systems.

Data Stream Category	Representative Biosurveillance System
Ambulance/EMT Records	Real-time Outbreak and Disease Surveillance (RODS) System
Clinic/Healthcare Provider Records	Electronic Surveillance System for the Early Notification of Community-Based Epidemics (ESSENCE)
ED/Hospital Records	Biosense 2.0
Employment/School Records	RODS, ESSENCE
Established Databases	Global Pest and Disease Database, World Animal Health Information Database, National Microbial Pathogen Database Resource
Financial Records	RODS
Help Lines	FirstWatch
Internet Search Queries	Google Flu Trends
Laboratory Records	ESSENCE
News Aggregators	HealthMap
Official Reports	CDC Reports, Ministry of Health Reports
Police/Fire Department Records	N/A
Personal Communication	Program for Monitoring Emerging Diseases (ProMed)
Prediction Markets	Iowa Health Prediction Market
Sales	National Retail Data Monitor (NRDM)
Social Media	Twitter

Two members of our team independently reviewed the documentation of the surveillance systems for information regarding the properties of the data streams and applied the concrete indicators developed for the metrics to derive values for use in the analysis. These values were then placed into a matrix (Table 8) that contained the value assigned to each data stream for each metric. If there were differences in the two independent reviews, a consensus was built through detailed discussions and gathering evidence base for the values.

**Table 8:** Matrix of Values for Data Streams.

	Accessibility	Cost	Credibility	Flexibility	Geo./Pop. Coverage	Granularity	Integrability	Specificity of Detection	Sustainability	Time to Indication	Timeliness
**Ambulance/EMT Records**	Medium	Medium	Medium	High	Global	Individual	Extremely	Low	Yes	Medium	Fast
**Clinic/Healthcare Provider Records**	Medium	Medium	High	High	Global	Individual	Extremely	High	Yes	Medium	Fast
**ED/Hospital Records**	Medium	Medium	High	High	Global	Individual	Extremely	High	Yes	Medium	Fast
**Employment/School Records**	Medium	Medium	Medium	Low	Global	Community	Moderately	Low	Yes	Indirect	Fast
**Established Databases**	Easy	Low	Low	High	Global	Community	Highly	Indirect	Yes	Long	Slow
**Financial Records**	Medium	Medium	Medium	Medium	Regional	Community	Moderately	Indirect	Yes	Long	Intermediate
**Help Lines**	Medium	Medium	Medium	Medium	Local	Community	Moderately	Medium	Yes	Near Real Time	Fast
**Internet Search Queries**	Easy	Low	Medium	High	Global	Community	Moderately	Medium	Yes	Near Real Time	Near Real Time
**Laboratory Records**	Medium	Medium	High	Medium	Global	Individual	Highly	High	Yes	Medium	Fast
**News Aggregators**	Easy	Low	Low	High	Global	Community	Moderately	Low	Yes	Near Real Time	Near Real Time
**Official Reports**	Easy	Medium	High	High	Global	Community	Moderately	High	Yes	Long	Intermediate
**Personal Communication**	Easy	Medium	Medium	High	Global	Individual	Not Very	High	Yes	Long	Fast
**Police/Fire Department Records**	Difficult	Medium	Low	Low	Global	Individual	Moderately	Indirect	Yes	Medium	Fast
**Prediction Markets**	Difficult	High	Low	Low	Global	Regional	Moderately	Medium	No	Indirect	Fast
**Sales**	Medium	Medium	Low	High	Regional	Community	Moderately	Low	Yes	Medium	Fast
**Social Media**	Easy	Low	Low	High	Global	Individual	Moderately	Low	Yes	Near Real Time	Near Real Time

## Results and Discussion

The results presented in this section are primarily to illustrate the application of the MAUT evaluation framework. There are several caveats to the assigned values for data streams as well as the assigned weights to the metrics that are being further investigated.

The purpose of MAUT is not to serve as a decision maker but, rather, is to inform and support the decision making process. The decision maker should use this prioritized list to inform their thought process and to help make justifiable and transparent decisions.The utility values determined for each of the data streams can be used to create a prioritized list of options.

**Figure 1:**
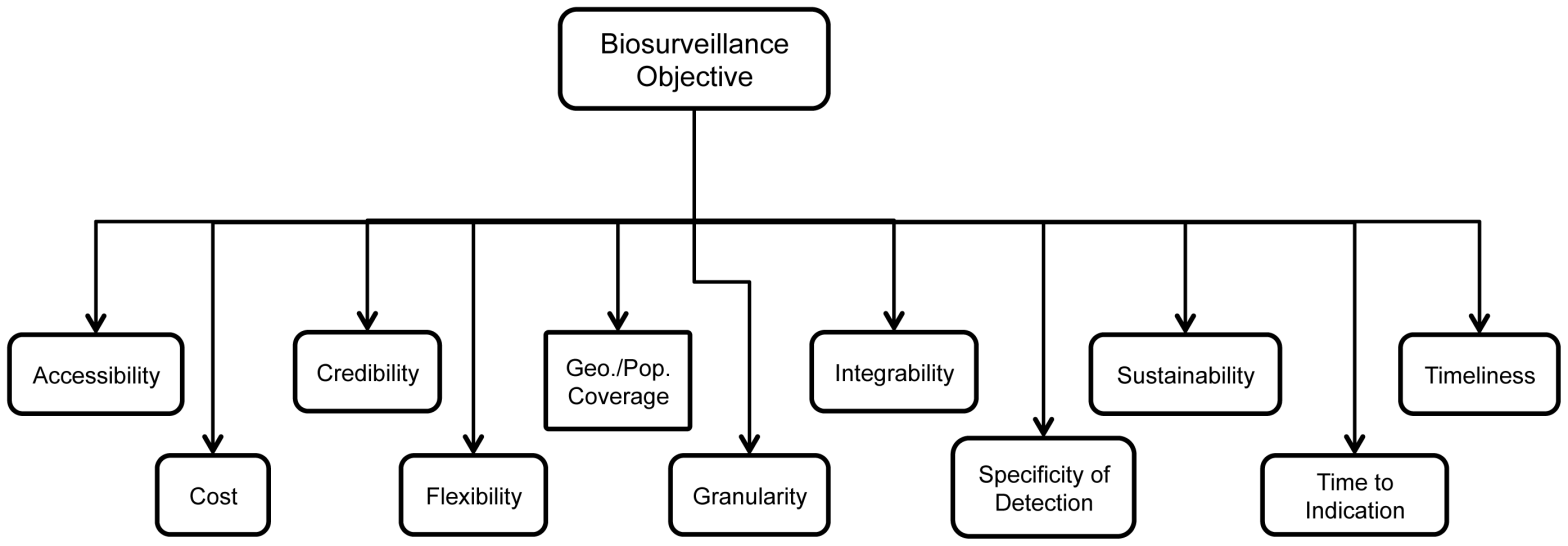
Example of objective hierarchy.

Data stream ranking was performed through the development of objectives hierarchies, a value tree that describes the hierarchy between the metrics and objectives. As the prioritizationof the metrics is dependent on the context of the biosurveillance objective, we had to design four hierarchies—one for each goal. While the hierarchies were the same for each, the objective specified and, thus, the metric weightswere different (Figure 1).

Following input of weights for metrics, values for each data stream for each metric and a single utility function for these values, the LDW tool generated four ranked lists of data streams, one for each surveillance goal, shown in Table 9.

**Table 9:** Ranking of Data Streamsby Biosurveillance Goal.

Data Stream	Early Warning of a Health Threat	Early Detection of a Health Event	Situational Awareness	Consequence Management
**Internet Search Queries**	1	1	3	3
**ED/Hospital Records**	2	2	1	1
**Clinic/Healthcare Provider Records**	2	2	1	1
**Laboratory Records**	3	3	2	2
**News Aggregators**	4	4	6	6
**Help Lines**	5	7	9	7
**Social Media**	6	5	7	6
**Ambulance/EMT Records**	7	6	5	5
**Personal Communication**	8	8	4	4
**Official Reports**	9	7	3	2
**Sales**	10	3	11	9
**Police/Fire Department Records**	11	10	12	10
**Employment/School Records**	12	11	8	8
**Financial Records**	12	12	10	9
**Established Databases**	13	13	12	11
**Prediction Markets**	14	14	13	12

Across the four biosurveillance objectives, there was a dichotomy exhibited between data streamcategory ranks in the early warning/early detection objectives and the situational awareness/consequence management objectives. As observed in Table 9, the ranks for the data streams are fairly consistent within the early warning/early detection goals and within the situational awareness/consequence management objectives. This seems to suggest that while we identified four distinct biosurveillance goals, functionally from the metric weights applied, there may only be two: pre/early event (i.e. the initial stages of an outbreak) and post-event.

Ranking results are a direct consequence of the values we assigned for each broad data stream category (as described in the methods), therefore, these results cannot be applied to specific data streams. Evaluation of specific data streams would invariably lead to differences in assigned values and would be reflected in the ranks attained. By generalizing, however, we were able to build a framework that could be easily applied to specific data streams.Four categories consistently ranked within the top five for every single goal: Internet Search Queries, ED/Hospital Records, Clinic/Healthcare Provider, and Laboratory Records. Three of these—ED/Hospital Records, Clinic/Healthcare Provider, and Laboratory Records—are commonly used in current systems; only Internet Search Queries are underutilized used as a data stream in operational biosurveillance systems.However, given how new Internet Search Queries are, it is not entirely unexpected and it may take time before this data stream category is adopted as a reliable source in systems. In the next level four data stream categories ranked consistently high among the similar goals (early warning/detection vs. situational awareness/consequence management): Official Reports, Personal Communication, News Aggregators, and Ambulance/EMT records. Official Reports ranked quite high for both situational awareness and consequence management, primarily due to the high values assigned for credibility and specificity of detection.

Social Media, Help Lines, and Sales data streams were all ranked at least once amongst the top five. After these data stream categories, there was a significant drop off in the ranks. In particular, five data streamscategorieswere consistently identified as being the least useful: Financial Records, Established Databases, Prediction Markets, Employment/School Records and Police/Fire Department Records. It is important to note that while certain data stream categories ranked low, it does not mean they are useless. It only means that for assigned values and the metric weights for this categorization ranked them low. Specific data streams that might have different values and weights could be evaluated differently depending on how the problem is being framed. For example, data stream categories such as Financial Records and Established Databases may be very useful when used together with more highly ranked data streams but given the limitations of this approach; it was not possible to take potential synergy of data streams into account.

The rankings of three data stream categories—Personal Communications, AmbulanceRecords, and News Aggregators—did not align with the experiences of several biosurveilance practitioners. While ranked highly for the situational awareness and consequence management goals, Personal Communication ranked towards the middle for the early warning and early detection goals. However, this data stream category was often cited byepidemiologists and biosurveillance practitioners as being one of the most important data streams they utilized to detect outbreaks in its early stages and to monitor its progress. Personal communications tend to be informal, highly unique and diverse in nature making it difficult to assign attributes using our approach—analysis by categories of data streams. A better understanding of the nature of these informal personal communication networks and what roles they may play in the decision making process leading up to an outbreak declaration may lead to some valuable insights that may lead to possible incorporation into future models.

Similarly, Ambulance Records ranked highly for both the Situational Awareness and Consequence Management goals. This result also did not align with the experiences of biosurveillance practitioners who described this data stream category as being highly useful for Early Warning. News Aggregators, while ranked highly for the Early Detection and Early Warning Goals, were deemed more useful for the other BSV goals by practitioners It is possible that the discrepancies in utility seen between the MAUT method and individual opinion is due to the fact that individuals may be inherently biased and may not take into account the multiple metrics that are considered in MAUT.

### Sensitivity Analysis

Given the highly customizable nature of MAUT, it was important to scope the problem and be able to obtain a defensible set of rankings for the data stream categories. The concept of “garbage in, garbage out” is equally as applicable to MAUT as it is to the field of computer science. Without properly structuring the problem and if poor data input choices are made, the output of the analysis is meaningless. The LDW tool as well as MCDA as a whole relies heavily on user input and customization and the rankings reported in this study may be influenced by the input parameters. It was important to make sure that the framework was robust as reflected in the stability of the rankings against variations in parameters. Sensitivity analysis was conducted by varying the dependent variables to understand their influence on data stream rankings. It is important to note that all changes for eachstrategy were applied simultaneously rather than looking at the effect of one variable sequentially, in order to maintain a realistic scope for the number of sensitivity analyses. The following strategies were used for this analysis:

1. Varying the utility function that describes the relationship between metric value and utility. By varying the utility function, it is possible to assess the impact of our assumptions on the relationship between the metric value and utility.
2. Varying weights of metrics; changing the weights in two ways assessed the impacts of the metric weights. The first was to set all metric weights equally so that each metric contributed to the final utility score. The second was to group the rankings of the metrics into three tiers (Table 10).
3. Performing rankings without Geographic/Population metric; for each data stream with the exception of three—Financial Records, Sales, and Help Lines—Geographic/Population coverage was uniformly assigned a value of “Global”. To see what impact this metric had on the final rankings, the rankings were recomputed without the Geographic/Population coverage metric.
4. Changing the most variable metric values in the matrix; we assigned values to data streams for each metric, using representative biosurveillance resources that routinely used specific data streams. To examine the influence of variable values on the final ranking of data stream categories, we ran Logical Decisions with an input of all low values for the data streamsthat showed most variability in certain metrics because in all cases, the final run utilized the higher values. This may also test the effect of individual biases.

**Table 10:** Metric Weights if Grouped into 3 Tiers.

Early Warning of a Health Threat		Early Detection of a Health Event		Situational Awareness		Consequence Management	
0.155	Specificity of Detection	0.161	Specificity of Detection	0.161	Geo./Pop. Coverage	0.145	Geo./Pop. Coverage
0.155	Credibility	0.161	Credibility	0.161	Credibility	0.145	Credibility
0.155	Time to Indication	0.161	Time to Indication	0.161	Time to Indication	0.145	Time to Indication
0.155	Timeliness	0.161	Timeliness	0.161	Timeliness	0.145	Timeliness
0.072	Flexibility	0.078	Geo./Pop. Coverage	0.078	Specificity of Detection	0.145	Specificity of Detection
0.072	Geo./Pop. Coverage	0.078	Granularity	0.078	Accessibility	0.078	Granularity
0.072	Granularity	0.078	Accessibility	0.078	Sustainability	0.078	Accessibility
0.072	Accessibility	0.03	Flexibility	0.03	Flexibility	0.03	Flexibility
0.03	Cost	0.03	Cost	0.03	Cost	0.03	Cost
0.03	Integrability	0.03	Integrability	0.03	Integrability	0.03	Integrability
0.03	Sustainability	0.03	Sustainability	0.03	Granularity	0.03	Sustainability

Tables 11–14 show the comparison of rankings obtained following sensitivity analysis, for each of the four biosurveillance goals. Overall, with the different sensitivity analyses, the results of the modified rankings suggest that the results obtained in the final rankings are robust. The same data streams that tend to be ranked as being most useful remain the top ranked. Similarly, the same data streams that tend to be ranked in the middle and at the bottom in the final rankings are observed to do the same in the modified rankings. Also, overall, there were few rises in rankings amongst the data streams across the different biosurveillance objectives.

**Table 11:** Comparison of Data Stream Rankings for Early Warning Surveillance Goal.

Early Warning of a Health Threat	Final Rankings	Without Geo./Pop. Coverage	Varying the Utility Function	3 Tiers of Metric Weights	Low Values for Metrics	Equal Weights	Highest Rank	Lowest Rank
ED/Hospital Records	2	2	1	1	3	1	1	3
Clinic/Healthcare Provider	2	2	1	1	3	1	1	3
Laboratory Records	3	4	2	3	5	3	2	5
Internet Search Queries	1	1	3	2	1	2	1	3
Official Reports	9	8	11	7	8	7	7	11
Personal Communication	8	7	9	4	7	6	4	9
Social Media	6	5	7	6	4	5	4	7
News Aggregators	4	4	5	5	2	4	2	5
Ambulance/EMT Records	7	6	4	9	6	5	4	9
Help Lines	5	3	6	8	3	8	3	8
Sales	10	9	8	10	9	9	8	10
Employment/School Records	12	12	12	11	11	10	10	12
Police/Fire Department Records	11	10	10	12	10	12	10	12
Financial Records	12	11	15	12	13	11	11	15
Established Databases	13	13	14	13	12	8	8	14
Prediction Markets	14	14	13	14	14	13	13	14

**Table 12:** Comparison of Data Stream Rankings for Early Detection Surveillance Goal.

Early Detection of a Health Events	Final Rankings	Without Geo./Pop. Coverage	Varying the Utility Function	3 Tiers of Metric Weights	Low Values for Metrics	Equal Weights	Highest Rank	Lowest Rank
ED/Hospital Records	2	2	1	1	2	1	1	2
Clinic/Healthcare Provider	2	2	1	1	2	1	1	2
Laboratory Records	3	3	2	2	4	3	2	4
Internet Search Queries	1	1	3	3	1	2	1	3
Official Reports	7	7	11	4	6	7	4	11
Personal Communication	8	7	9	5	7	6	5	9
Social Media	5	5	6	7	3	5	3	7
News Aggregators	4	4	5	6	2	4	2	6
Ambulance/EMT Records	6	6	4	8	5	5	4	8
Help Lines	7	5	7	9	6	8	5	9
Sales	9	8	8	10	8	9	8	10
Employment/School Records	11	11	12	11	10	10	10	12
Police/Fire Department Records	10	9	10	12	9	12	9	12
Financial Records	12	10	15	13	11	11	10	15
Established Databases	13	12	14	14	12	8	8	14
Prediction Markets	14	13	13	15	13	13	13	15

**Table 13:** Comparison of Data Stream Rankings for Situational Awareness Surveillance Goal.

Situational Awareness	Final Rankings	Without Geo./Pop. Coverage	Varying the Utility Function	3 Tiers of Metric Weights	Low Values for Metrics	Equal Weights	Highest Rank	Lowest Rank
ED/Hospital Records	1	1	1	2	1	1	1	2
Clinic/Healthcare Provider	1	1	1	2	1	1	1	2
Laboratory Records	2	2	2	3	3	3	2	3
Internet Search Queries	3	2	3	1	2	2	1	3
Official Reports	3	3	4	5	2	7	2	7
Personal Communication	4	4	6	7	4	6	4	7
Social Media	7	8	8	6	7	5	5	8
News Aggregators	6	7	7	4	6	4	4	7
Ambulance/EMT Records	5	6	5	7	5	5	5	7
Help Lines	9	5	11	8	9	8	5	11
Sales	11	9	9	9	10	9	9	11
Employment/School Records	8	9	9	9	8	10	8	10
Police/Fire Department Records	12	11	9	10	11	12	9	12
Financial Records	10	10	13	11	12	11	10	13
Established Databases	12	12	10	12	11	8	8	12
Prediction Markets	13	13	12	13	13	13	12	13

**Table 14:** Comparison of Data Stream Rankings for Consequence Management Goal.

Consequence Management	Final Rankings	Without Geo./Pop. Coverage	Varying the Utility Function	3 Tiers of Metric Weights	Low Values for Metrics	Equal Weights	Highest Rank	Lowest Rank
ED/Hospital Records	1	1	1	1	1	1	1	1
Clinic/Healthcare Provider	1	1	1	1	1	1	1	1
Laboratory Records	2	2	2	3	2	3	2	3
Internet Search Queries	3	3	4	2	3	2	2	4
Official Reports	3	4	3	4	3	7	3	7
Personal Communication	4	5	5	5	4	6	4	6
Social Media	6	8	8	7	6	5	5	8
News Aggregators	6	8	7	6	6	4	4	8
Ambulance/EMT Records	5	7	6	8	5	5	5	8
Help Lines	7	6	9	9	7	8	6	9
Sales	9	10	10	10	9	9	9	10
Employment/School Records	8	9	12	10	8	10	8	12
Police/Fire Department Records	10	12	11	11	10	12	10	12
Financial Records	9	11	15	12	13	11	9	15
Established Databases	11	13	14	12	11	8	8	14
Prediction Markets	12	14	13	13	12	13	12	14

Through the development of an evaluation framework for determining the utility of biosurveillance data streams, and application of the MAUT tool to rank data streams, we have demonstrated aproof of principle for the application ofmulti-criteria decision analysis to the problem of data stream selection for biosurveillance systems. Thisuniversal evaluationframework offers biosurveillance practitioners a structured, methodological approach to evaluating data streams for inclusion into biosurveillance systems and forces systematic thinking. By employing MAUT, this framework seeks to address many of the shortcomings found in evaluations of biosurveillance systems. It is capable of evaluating multiple criteria, both qualitative and quantitative, thus allowing for a more comprehensive evaluation than if a single criterion were used. Additionally, MAUT is a flexible enough tool that can be configured to evaluate multiple types of biosurveillance data streams and can be configured to handle quantitative criteria, a feature we did not use. The danger in prescribing a single set of unvarying metrics or weights of metrics is that biosurveillance is a multi-faceted process and the metrics that may be useful for one surveillance goal may not be useful for another. This is extremely true as the target of surveillance changes species or disease type. The advantage of our framework is that it is species and disease agnostic and can be employed to evaluate the many types of data streams found in biosurveillance.

MAUT provides a systematic and structured methodology for biosurveillance practitioners presented with a complex decision—the selection of essential information. Most decision-making in the realm of public health or biosurveillance data stream evaluation has traditionally relied upon a highly subjective, ad hoc approach that favors intuition and personal experience or utilizes one or two quantitative metrics that are unable to capture the range of criteria needed to systematically evaluate data streams [22]. Part of the challenge is that people are “quite bad at making complex, unaided decisions” [23] and relying just on intuition or personal experience does not lead to better decisions [24]. Since most complex decisions require considering multiple metrics, including non-quantitative ones, a method that can incorporate both is essential. Simply relying upon the expertise of an individual or a small group of individuals will not, alone, address the common shortfalls currently in practice in the face of complex decision making. MAUT additionally provides biosurveillance practitioners with an open, explicit, and defensible approach that can also serve as an audit trail.

Using a MAUT framework and approach provides a formal technique that can assess both quantitative and qualitative metrics (which are characteristics of complex decisions), thereby providing the decision maker with an methodology that can deconstruct the complex decision into multiple, more manageable pieces, allow data and judgment to be made to the smaller pieces, and then to reconstruct the pieces into a more complete picture of the problem for the decision maker.

While the MAUT approach offers a systematic and objective approach to evaluating data streams, there are inherent limitations to this approach that must be carefully considered and accounted for when interpreting the results of such an analysis.

MAUT is heavily data driven and requires significant user input to structure the problem and elicit the values, and if this input is inaccurate, the results can be of little value. Facilitating an accurate analysisthrough rigorous stakeholder elicitation can be both time consuming and expensive. Because of this heavy dependence on user input, MAUT is sensitive to omitted or inaccurate input. In the case of our application of the method and framework, this potential limitation is observed in howthe values to input for themetrics were determined. By focusing on using values and properties of data streams in use within a biosurveillance system that was representative of that type of data stream category, the results maybe biased towards more traditional data stream categories.Another limitation that also stems from the heavy user input dependence wasobserved in the non-representative group opinionwe used to elicit metric weights. In particular, the composition of this project's SME panel exhibited a bias towards experts in human health that represented an understanding of surveillance practiced predominantly within the developed world, and was largely academic. As a result, their opinions onmetric weights may not accurately align with operational practice. This in particular is observed by the near universal ranking of cost as being a metric of low importance, in spite of biosurveillance practitioners identifying it as one of the most important. This bias was additionallyobserved and supported by Gajweski et al. [25] who in the course of reviewing evaluationsof electronic event-based biosurveillance systems noted that the least frequently considered attribute was cost.

As MAUT treatseach metric independently, interdependencies amongst metrics cannot be taken into account—the interrelation between accessibility and cost being an example. Similarly, this framework does not consider the synergistic effects that may emerge when utilizing multiple,different but complementary data streams. For example, certain data streams, such as personal communications and established databases, lend themselves as being useful in a synergistic fashion. Historical climate data that can be used to establish baseline levels of weather, while not indicative of a disease outbreak directly, can be used to predict mosquito incidence. Both of these limitations are present in this evaluation of data streams. An additional limitation to MAUT is that it assumes that maximization of utility is the most important criteria in the decision making process which may not be true. For example, in some cases political factors may play a larger role to the decision maker than sheer maximization of utility. This project demonstrates a proof of principle for the application of MAUT to biosurveillance data stream evaluation, and hopes to build upon this to refine the use of MAUT.

The ranking of data streams served to illustrate the application of our evaluation framework. While we used a certain approach assigning values to data streams and weights to metrics, we acknowledge that this is by no means the best approach and that there may be several, better ways to do so. We hope to primarily convey the systematic and structural approach to thinking about how to select “essential information”.

In today's economic and political climate, there is even more of a need for evaluation of potential and current biosurveillance systems and data streams to ensure that limited financial and human resources are being effectively deployed. We have demonstrated the utility of an evaluation framework based on MAUT that can assist practitioners in their decision making process. This framework balancesthe complexity of biosurveillance data stream evaluation by minimizing the subjectivity of evaluation and by providing documentation that allows for increased transparency and consistency. This evaluation framework and MAUT model should be used with careful consideration to the sensitivity and robustness of results and should be seen not so much as a decision making tool but rather as a decision aid to supportthe prioritization and selection of data streams for specific biosurveillance goals.

## Supporting Information

Methods S1 **Methods of Determining Values of Metrics.**
(DOCX)
