# Supplementary material for: Selecting Essential Information for Biosurveillance—A Multi-Criteria Decision Analysis
Source: PLoS One. 2014 Jan 29;9(1):e86601. doi: 10.1371/journal.pone.0086601 (PMC3906072; doi:10.1371/journal.pone.0086601)
Supplement: Methods S1 — Methods of Determining Values of Metrics. (DOCX) [file pone.0086601.s001.docx]

**Supplementary Methods S1**

**Methods of Determining Values of Metrics**

Outlined below are the methods of how the values for the inputs for the metrics were determined. As each reviewer was going through the relevant literature to the data stream type they were evaluating, they would determine the input values into the metrics bases upon the performance of the data stream using the criteria outline below.

**Accessibility**
Definition: The extent to which the data stream is available.

Accessibility is measured as a label with three options: Difficult Accessibility, Medium Accessibility, and Easy Accessibility.

- ***Difficult Accessibility***- is when the data stream being analyzed has been used in at least ones system and faces many (3 or more) obstacles in data access
- ***Medium Accessibility***- is when the data stream being analyzed has been used in at least one system and faces some (less than 3) obstacles in data access
- ***Easy Accessibility***- is when the data from a particular data stream is freely accessible

The *utility* increases, the easier the accessibility.

Examples of obstacles include: privacy concern, passwords, subscription, membership/ group affiliation, non-digitized information, etc.

**Cost**

Definition: The costs to set-up, operate, and maintain the data stream

- ***High Cost***—there is a cost to obtain, set-up, and maintain the data stream
- ***Medium Cost***—there is a cost for only two of three of the following: to obtain, set-up, or maintain the data stream
- ***Low Cost***—there is a cost for only one of the following: to obtain, set-up, or maintain the data stream

The *utility* decreases the lower the cost.

**Credibility**

Definition: The extent to which the data stream is considered reliable and accurate

Credibility is measured as a label with three options: Low Credibility, Medium Credibility, and High Credibility.

- ***Low Credibility***- is when the data stream being analyzed provides limited actionable results. Additionally, data must be validated by another source
- ***Medium Credibility***- is when the data stream being analyzed provides actionable results but data still requires validation
- ***High Credibility***- is when the data stream being analyzed provides actionable results and minimal validation needed

The *utility* increases the higher the credibility.

An actionable result refers to when the data provided by the data stream is of high enough quality that it can be acted on. Validation refers to the need to confirm the data from the data stream using a separate source.

**Flexibility**

Definition: The data stream’s ability to be used for more than one purpose (such as for use in surveillance for more than one disease, or for more than one goal)

Flexibility is measured as a label with three options: High Flexibility, Medium Flexibility, and Low Flexibility.

- ***High Flexibility***- is when the data stream being analyzed can be used for more than three purposes
- ***Medium Flexibility***- is when the data stream being analyzed can be used for two purposes
- ***Low Flexibility***- is when the data stream being analyzed can be used for only one purpose

The *utility* increases for the more purposes the data stream can be used for.

Examples of purposes include: diseases, events, goals, types of surveillance etc.

**Integrability**

Definition: How well the data stream can be linked/combined with other data streams

Integrability is measured as a label with four options: Extremely Integrable, Highly Integrable, Moderately Integrable, and Not Very Integrable.

- ***Extremely Integrable***- is when the data from the data stream is in a structured and standardized format and has been integrated with one other data stream in more than one biosurveillance system
- ***Highly Integrable***- is when the data from the data stream is structured and in a standardized format and has been integrated with one other data stream in one other biosurveillance system
- ***Moderately Integrable***- is when the data from the data stream is either in a structured format or has been integrated with one other type of data
- ***Not Very Integrable***- is when the data from the data stream is unstructured and has never been integrated with another type of data.

The *utility* increases if the data stream is in a standardized format and if it has been integrated with other types of data.

Structured data implies xml or other electronic format. A standardized format implies ICD-9, or other agreed upon reporting formats. Integrated refers to the data from one data stream being combined or linked to the data in another data stream.

**Geographic/ Population coverage**

Definition: The geographic or population area of coverage

Geographic/ Population coverage is a label with four options: Local, Regional, National, and Global.

The *utility* increases as the Geographic and Population coverage becomes broader (i.e. more global)

**Granularity**Definition: The level of detail of the data stream

Granularity is measured as a label with four options: Individual, Community, Regional, and National.

- ***Individual-*** the data is applicable at the individual level
- ***Community***- the data is applicable at the community level
- ***Regional***- the data is applicable at the regional level
- ***National***- the data is applicable at the national level

The *utility* increases as the unit of data tracked becomes smaller.

An individual level refers to a person. A community level is anything from a household to a metropolitan area of a large city. A regional level refers to state or a state-like entity or a grouping of states.

**Specificity of Detection**

Definition: The ability of the data stream to identify an outbreak, event, disease, or pathogen of interest

Specificity of Detection is measured as a label with four options: High Specificity, Medium Specificity, Low Specificity, and Indirect Specificity.

- ***High Specificity***- the method of detection for the data stream is disease specific
- ***Medium Specificity***- the method of detection for the data stream is disease category specific (e.g. viral, bacterial, etc.)
- ***Low Specificity***- the method of detection for the data streams tells you syndrome-based information (e.g. ILI, etc.)
- ***Indirect Specificity***- the method of detection for the data stream is an indirect indicator of disease

The *utility* is highest for High Specificity and lowest for Indirect Specificity.

**Sustainability**

Definition: The data stream’s continued availability over time

Sustainability is measured as a label with two options: Yes and No.

***Yes-*** the data stream is still in use / existence

***No-*** the data stream is not still in use / existence

The *utility* is higher for data streams that are still in use / existence.

**Timeliness**

Definition: The time required for the data stream to first signal a disease, outbreak, or event

Timeliness is measured as a label with four options: Near Real Time, Quick, Intermediate, and Slow.

- ***Near Real Time***- The data is available within one day
- ***Fast***- The data is available between one day and one week
- ***Intermediate***- The data is available between one week and one month
- ***Slow***- The data is available after one month

The *utility* decreases the longer it takes for the data to become available.

**Time to Indication**

Definition: Earliest time that the data is available

Time to Indication is measured as a label with four options: No Indication, Near Real Time Indication, Medium Indication, and Long Indication.

- ***Long Indication***- The data stream indicates an event, situation or disease outbreak after one week
- ***Medium Indication***- The data stream indicates an event, situation or disease outbreak between one day and one week
- ***Near Real Time Indication***- The data stream indicates an event, situation or disease outbreak within a day.
- ***Indirect Indication***- The data stream does not directly indicate an event, situation, or disease outbreak

The *utility* decreases the longer it takes for the data stream to indicate an event, situation or disease outbreak.
